# Assessment of left and right ventricular trabeculation and non-compacted myocardium in a large selected healthy reference population

**DOI:** 10.1186/1532-429X-16-S1-P303

**Published:** 2014-01-16

**Authors:** Florian Andre, Astrid Burger, Dirk Lossnitzer, Sebastian Buss, Hassan Abdel-Aty, Evangelos Giannitsis, Henning Steen, Hugo A Katus

**Affiliations:** 1 Department of Cardiology, University of Heidelberg, Heidelberg, Germany

## Background

The differentiation of left ventricular non-compaction (LVNC) from physiological trabeculation is still challenging as it may be overdiagnosed by current criteria. Therefore, we sought to investigate the effect of age and gender on both the LV and RV trabeculation in a large group of proven healthy subjects to provide adjusted reference values. Furthermore, we examined the correlation between the amount of non-compacted myocardium and the global ventricular function.

## Methods

We studied a selected reference population of 117 healthy volunteers (58 male, 59 female) stratified into age tertiles (group 1 = 20-34 years, group 2 = 35-50 years, group 3 > 50 years) and by gender. Cardiac diseases were meticulously ruled out by taken medical history, physical examination, electrocardiography, oral glucose tolerance test and lab tests. Cardiovascular magnetic resonance images were acquired in a 1.5T whole-body MRI (Achieva, Philips Heathcare) using a standard SSFP sequence. End-diastolic (EDV), end-systolic (ESV), stroke and trabeculated volumes as well as ejection fraction (EF) were quantified in short-axis views from base to apex. Volumes were indexed to body surface area (BSA). The non-compacted-to-compacted (NC/C)-ratio was measured in 4-chamber view.

## Results

The left and right ventricular (LV/RV) trabeculated volumes were significantly larger in men than in women and decreased with age (LV: s = -0.1262, R^2 ^= 0.04450, p < 0.05; RV: s = -0.4067, R^2 ^= 0.2204, p < 0.001; see Table 1). They showed a negative relation to EF and a positive one to EDV and ESV (LV: r = -0.27, r = 0.74, r = 0.59, all p < 0.01; RV: r = -0.38, r = 0.65, r = 0.60; all p < 0.01). The correlation between LV and RV trabeculated volume was moderate (r = 0.46, p < 0.001). The NC/C ratio was > 2.3 in 28 of 117 subjects (23.9%) and > 2.5 in 26 of 117 subjects (22.2%), which is regarded as pathologic in current literature. The fraction of subjects with a NC/C ratio > 2.3 differed significantly between age but not between gender groups (p < 0.05, p = n.s). Likewise the mean NC/C ratio showed no significant differences between gender groups (1.9 ± 0.7 vs. 2.1 ± 0.8, p = n.s.) whereas subjects of the first tertile showed significant higher values than the volunteers of the second tertile (2.2 ± 0.8 vs. 1.8 ± 0.6, p < 0.05). An increasing NC/C ratio and LV trabeculated volume were associated with a significant decrease in EF (r = -0.24, r = -0.27, both p < 0.01).

**Table 1:** LV and RV trabeculated volumes

	LV trabeculated volume/BSA			RV trabeculated volume/BSA		
	**male**	**female**		**male**	**female**	

Group I	45.6 ± 8.3	40.7 ± 6.0	p < 0.05	51.8 ± 11.6	44.4 ± 7.8	p < 0.05

Group II	43.8 ± 10.2	36.4 ± 4.4	p < 0.01	42.3 ± 10.4	32.7 ± 6.6	p < 0.01

Group III	39.8 ± 6.4	37.1 ± 6.4	p = n.s	39.2 ± 8.0	31.8 ± 9.0	p < 0.05

	43.1 ± 8.7	38.1 ± 5.9	p < 0.01	44.6 ± 11.4	36.4 ± 9.6	p < 0.01

Volumes are given in ml/m^2^.

## Conclusions

A considerable fraction of healthy volunteers fulfill the current diagnostic criteria for LVNC. The amount of trabeculated volume depends on age and gender so that the use of age- and gender-specific reference values as provided in this study may facilitate the delineation of physiological and pathological findings. In addition, the trabeculated volume contributes below average to the EF even in healthy volunteers. Further studies are necessary to investigate the clinical impact of this finding.

## Funding

None.

